# Regulatory T-Cell Development in the Human Thymus

**DOI:** 10.3389/fimmu.2015.00395

**Published:** 2015-08-03

**Authors:** Íris Caramalho, Helena Nunes-Cabaço, Russell B. Foxall, Ana E. Sousa

**Affiliations:** ^1^Instituto de Medicina Molecular, Faculdade de Medicina, Universidade de Lisboa, Lisbon, Portugal

**Keywords:** human thymus, regulatory T-cells, FOXP3, regulatory T-cell development, human thymic defects, primary immunodeficiency

## Abstract

The thymus generates a lineage-committed subset of regulatory T-cells (Tregs), best identified by the expression of the transcription factor FOXP3. The development of thymus-derived Tregs is known to require high-avidity interaction with MHC-self peptides leading to the generation of self-reactive Tregs fundamental for the maintenance of self-tolerance. Notwithstanding their crucial role in the control of immune responses, human thymic Treg differentiation remains poorly understood. In this mini-review, we will focus on the developmental stages at which Treg lineage commitment occurs, and their spatial localization in the human thymus, reviewing the molecular requirements, including T-cell receptor and cytokine signaling, as well as the cellular interactions involved. An overview of the impact of described thymic defects on the Treg compartment will be provided, illustrating the importance of these *in vivo* models to investigate human Treg development.

## Introduction

Regulatory T-cells (Tregs) play a major role in immune homeostasis by preventing or limiting T-cell activation, particularly in the context of auto-antigens. Expression of the transcription factor forkhead box P3 (FOXP3), considered a master regulator of Treg development and function, is essential for their role in the maintenance of dominant tolerance [reviewed in Ref. (1)]. Conditions where FOXP3 is defective or absent, such as the recessive disorder immune dysregulation, polyendocrinopathy, enteropathy, X-linked (IPEX) syndrome, are characterized by aggressive autoimmune manifestations that are usually fatal within the first 2 years of life, unless corrected via hematopoietic stem-cell transplantation (2–4).

Regulatory T-cells develop primarily in the thymus (thymus-derived Tregs, tTregs), although they can also be differentiated in the periphery (peripherally-induced Tregs). The delineation of these two populations in the peripheral Treg compartment is difficult due to the lack of specific markers. Nevertheless, tTregs are thought to be enriched in self-reactive T-cell receptors (TCRs) and to be critical for the maintenance of self-tolerance [reviewed in Ref. (1)]. Despite extensive research on tTreg development using murine models in the past 20 years, many questions remain unanswered regarding the mechanisms involved in the establishment and maintenance of the tTreg lineage [reviewed in Ref. (5, 6)]. Additionally, human tTreg studies are constrained by the limited number of tools available. Data have been mainly generated by *in vitro* manipulation of human thymic tissue or *in vivo* models using mouse/human chimeras [reviewed in Ref. (7)].

In this regard, the information provided by genetic human diseases characterized by thymus-related disturbances, such as the DiGeorge, the Omenn, and the autoimmune polyendocrinopathy candidiasis ectodermal dystrophy (APECED) syndromes, have been instrumental [reviewed in Ref. (8, 9)]. DiGeorge syndrome features athymia in <1% of the patients, but in the majority of them the prevailing thymic hypoplasia is associated with mild to moderate T-cell lymphopenia, and increased incidence of infection and autoimmune diseases [reviewed in Ref. (10)]. Alterations in the circulating Treg compartment have been consistently reported in DiGeorge syndrome (11–13). Notably, a decreased proportion and number of FOXP3^+^ cells in pediatric patients older than 2 years was observed when compared to age-matched healthy controls, in direct correlation with thymic output, as estimated by the numbers of CD4^+^ recent thymic emigrants (RTEs, defined by the expression of CD31 and CD45RA) (12). Omenn syndrome results from hypormorphic mutations in severe combined immunodeficiency (SCID)-causing genes and is associated with the generation of a limited pool of T lymphocytes with a restricted repertoire and activated phenotype [reviewed in Ref. (8)]. Loss of corticomedullary junction and Hassall’s bodies with depletion of autoimmune regulator gene (AIRE)-expressing medullary thymic epithelial cells (mTECs) and thymic dendritic cells (DCs) were described in two Omenn patients, in parallel with a dramatic decrease of Tregs in the thymus (14), supporting a role of AIRE-expressing mTEC and/or thymic DCs in their differentiation. In agreement, loss-of-function mutations in the *AIRE* gene (APECED syndrome) have been linked to a defective circulating Treg compartment (15–17). Not only is the frequency of naïve/resting Tregs (defined as CD4^+^FOXP3^+^CD45RO^neg^CD31^+^) decreased in APECED patients but also their levels of FOXP3 expression, function, and repertoire are altered, further supporting an abnormal tTreg development in the absence of AIRE (16).

A recent study that involved a paired analysis of thymic and blood samples in young children (newborns to 1-year-old) showed a direct correlation between the size of the two Treg compartments, further supporting the importance of the thymus for the establishment of the peripheral Treg pool early in life (18). Of note, both human and murine RTEs are endowed with enhanced potential to convert into peripherally-induced Tregs, when compared to their more mature counterparts, implying an additional role of the thymus for the setting of the peripheral Treg compartment (19). Furthermore, patients with athymia due to complete DiGeorge or FOXN1 deficiency have been shown to recover the peripheral Treg compartment upon allogeneic thymus transplantation, irrespective of the degree of HLA mismatching (20–22).

It is vitally important to understand human tTreg development in order to devise strategies to manipulate their generation as well as their repertoire (23). This mini-review will provide an overview of the current knowledge regarding human tTreg development, as well as the fundamental questions that remain to be addressed.

## Commitment to the Treg Lineage: When?

The human thymic primordium is colonized by T-cell progenitors during the 8th week of gestation, but mature T-cells are only observed in the thymus at the 12th to 13th gestational weeks (24–26). At this stage, human tTregs can already be found in the thymus (27–29). The frequency of fetal human tTregs, identified by their elevated expression of the high-affinity IL-2 receptor alpha chain (IL-2Rα/CD25), was found to be stable throughout gestation (representing 6–7% of total thymocytes) and similar to the proportion observed in infant thymuses (28).

Fetal human tTregs already express FOXP3, as assessed at the gene expression level, as well as other markers related to their suppressive phenotype, such as cytotoxic T-lymphocyte-associated protein 4 (CTLA-4) and glucocorticoid-induced TNFR-related protein (GITR) (27, 28). Moreover, these fetal human tTregs have the ability to suppress T-cell proliferation (27, 28).

One question that has been extensively addressed in mice, but due to technical limitations only in a few human studies, is when and how a thymocyte becomes committed to the Treg lineage. The discrete populations that express Treg markers, such as CD25 and FOXP3, in the human post-natal thymus mainly comprise mature CD4 single-positive (CD4SP, CD4^+^CD8^neg^) thymocytes, but also include CD8 single-positive (CD8SP, CD4^neg^CD8^+^) and double-positive (DP, CD4^+^CD8^+^) thymocytes, as well as cells in early pre-DP stages (27, 28, 30–36). In agreement, FOXP3^+^/CD25^+^ thymocytes can be found mostly in the medullary region of the human thymus, where mature thymocytes localize, with rare cells scattered in the cortex (30, 31, 35, 37, 38). The mechanisms that allow for human tTreg commitment to occur are still ill-defined.

We and others have reported pre-DP expression of FOXP3, namely, at the triple-negative and CD4 immature single-positive stages (33, 35) (Table 1). However, the contribution of this population to the human tTreg pool remains to be addressed.

Double-positive thymocytes expressing FOXP3 and/or CD25 are clearly identified in the human thymus. They additionally express other Treg function-associated markers, such as CTLA-4, CD39, and GITR (27, 28, 36), and exhibit suppressive function (32, 36) (Table 1). DP tTregs feature some degree of immaturity, as evidenced by the expression of recombination-activating gene 2 mRNA (34). Moreover, upon stripping of surface molecules using pronase, DP tTregs re-acquire both CD4 and CD8 at the surface, confirming their bona-fide DP status (36). Nevertheless, the majority of human DP tTregs express high levels of CD3 and CD27, which are associated with positive selection and maturity (27, 36) (Table 1). Importantly, DP tTregs are thought to significantly contribute to the CD4SP tTreg pool in humans, as predicted by linear regression models (36), and formally demonstrated by co-cultures of DP thymocytes with either TEC (36) or mature plasmacytoid (p)DCs (38). This observation contrasts with what has been described in murine models, where Foxp3 induction, although possible at the DP stage (45–47), mostly occurs at the CD4SP stage (48). Of note, human CD4SP CD25^neg^ thymocytes are also permissive to FOXP3 acquisition (37, 39, 49).

CD4SP tTreg represent the major population of FOXP3^+^ human thymocytes. They phenotypically mirror peripheral Tregs and exhibit efficient regulatory function (27, 28, 30, 36, 41, 43) (Table 1). The contribution of recirculating peripheral Tregs to this tTreg compartment is still debatable. It has long been proposed that activated T-cells may recirculate back to the thymus (50, 51), although this issue is particularly difficult to assess in humans. Recently, it was reported that a considerable proportion of human CD4SP tTregs may consist of recirculating cells (44). This was based on the observation that approximately one-fourth of these cells had lost CD31 and acquired ICOS and Tbet expression. Although it is not possible to exclude that a fraction of human tTregs may actually represent mature recirculating cells, there are some caveats to this interpretation. For instance, ICOS is already expressed by tTreg at the DP stage (39). Also, the high FOXP3 expression levels found within CD4SP ICOS^+^CD31^neg^ tTregs (44) may reflect their interaction with ICOSL expressed on mTECs, as previously described (52). Moreover, TREC levels are reportedly comparable between the CD4SP CD25^+^ and CD25^neg^ thymocyte populations, and several logs higher that those found in circulating Tregs, supporting that the majority of CD4SP tTregs are at the final stage of T-cell development (53). Of note, Vbeta usage and spectratyping analyses supported that CD4SP tTregs and CD4SP FOXP3^neg^/CD25^neg^ thymocytes have a similarly diverse repertoire (36, 53). To our knowledge, the direct comparison of the thymic CD4SP FOXP3^+^ and FOXP3^neg^ repertoires has not been reported. Its assessment will be important to clarify this issue since in peripheral cells the Treg repertoire has only 24% overlap with conventional CD4 T-cells (54). Additional studies will be instrumental in determining the magnitude and role of mature Treg recirculation in the human thymus.

**Table 1:** **Characterization of human post-natal thymic Tregs**.

Markers	Pre-DP	DP	CD4SP	CD8SP	Reference
FOXP3	+	+++	+++	++	(31, 33, 35, 36, 39, 40)
CD25	−	+++	+++	++	(30, 31, 33, 35, 36, 39, 41)
CTLA-4	+	+++	+++	++	(30, 31, 33, 36, 39, 41, 42)
CD127	−/+	+	−	−	(33–36)
HLA-DR	ND	++	+	+	(36)
CD39	ND	++	++	+	(36)
CD73	ND	−	−	+	(36)
CD103	ND	+	−	++	(36)
ICOS	ND	++	++	+	(39, 43, 44)
CD69	ND	++	++	+	(34, 36, 42)
CD27	ND	++	++	++	(36, 42)
Ki67	+	+	−/+	−/+	(36)
Suppressive capacity	ND	Yes	Yes	Yes	(30–32, 36, 39, 41)

*ND, not determined*.

Thus, tTreg lineage commitment may occur at various stages of human T-cell development.

## Commitment to the Treg Lineage: How?

Studies in mice have clearly established the requirement for TCR stimulation in Treg lineage commitment [reviewed in Ref. (5, 6)]. In humans, technical limitations preclude a direct assessment of the role of TCR signaling in tTreg development. We and others have shown that human tTreg differentiation is associated with markers of positive selection, such as CD69 and CD27 (27, 28, 34, 36, 42) (Table 1). Moreover, binding sites for the TCR downstream targets NFAT and AP1 are present within the human *FOXP3* promoter that are directly activated by TCR stimulation (55). Notably, both DP and CD4SP tTreg express CTLA-4 (30, 36), a molecule that in mice was shown to be downstream of Nur77, an immediate early gene upregulated by TCR stimulation (56). Indirect evidence that enhanced TCR signaling strength may dictate thymocyte commitment into the Treg lineage can be further inferred by the increase in CD4SP CD25^+^FOXP3^+^ thymocyte number in humanized mice treated with a superagonist anti-CD28 mAb (57). Moreover, human ZAP70-deficient patients present a dramatic decrease in the frequency and number of tTregs (58). Interestingly, CD4SP CD25^+^ tTregs were shown to frequently express two functional TCRs, in association with enhanced FOXP3 expression, suggesting that dual TCR expression may favor tTreg lineage commitment in humans (40). Overall, available evidence support that TCR signaling strength guides thymocyte commitment into the Treg lineage in humans, with a predicted impact on their self-reactivity.

Several additional signaling pathways and molecular factors have been implicated in human tTreg differentiation and/or proliferation, namely JAK3/STAT-5, Notch, CD80/CD86, ICOS/ICOSL, CD40/CD40L, thymic stromal lymphopoietin (TSLP), as well as the common-gamma chain (γC) cytokines interleukin (IL)-2 and -15 (37–39, 49, 52, 59). Watanabe et al. demonstrated that TSLP from Hassall’s bodies activate myeloid (m)DCs, enabling them to induce tTreg differentiation from CD4SP CD25^neg^ thymocytes (37). pDCs are also capable of driving CD4SP non-regulatory thymocytes into the human Treg lineage, upon response to TSLP (49). Cognate interactions between mDCs or pDCs and CD4SP non-regulatory thymocytes are required, as differentiation was impaired by HLA-DR blockade (37, 49). Activation of pDCs with anti-CD40L and IL-3 also confers on them the capacity to differentiate post-selection DP CD69^hi^TCR^hi^ thymocytes into human tTregs (38). Additionally, mTECs were shown to promote the survival and proliferation of human tTregs in an ICOSL-dependent mechanism that required the presence of conventional CD4SP cells as source of IL-2 (52). We have recently investigated the requirement of γC cytokines in human tTreg development and established a critical role for both IL-2 and IL-15 in their lineage commitment, as well as in tTreg proliferation and survival post-selection (39). This study also allowed the identification of macrophages and B lymphocytes as main IL-15 producers that likely represent two additional thymic antigen-presenting cell populations involved in human tTreg differentiation (39). In agreement, we found FOXP3^+^ cells in close vicinity of both macrophages and B cells in the human thymus (39). Accordingly, B lymphocytes were recently shown to be capable of selecting tTreg in mice (60, 61), and the majority of human CD4SP tTregs were shown to express CCR8 endowing them with the capacity to migrate in response to chemokines produced by macrophages (30).

**Figure 1:**
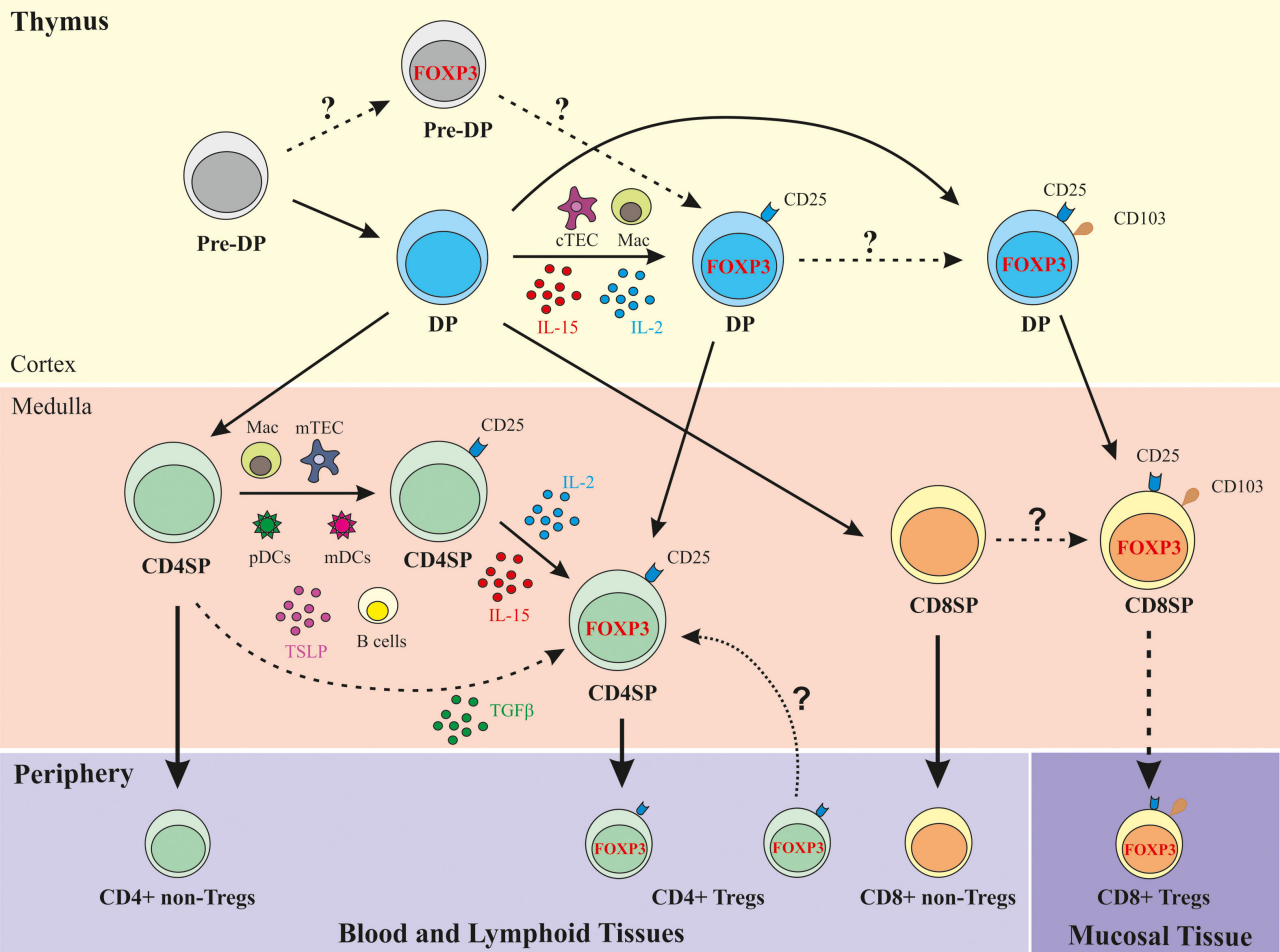
**Schematic representation of human Treg development in the human thymus**. DP, double-positive (CD4^+^CD8^+^); CD4SP, CD4 single-positive (CD4^+^CD8^neg^); CD8SP, CD8 single-positive (CD8^+^CD4^neg^); cTEC, cortical thymic epithelial cell; mTEC, medullary TEC; Mac, macrophage; FOXP3, Forkhead box P3; TSLP, thymic stromal lymphopoietin.

As illustrated in Figure 1, the most immature thymocyte population that clearly expresses FOXP3, in addition to other Treg function-associated markers, such as CD25, CTLA-4, and CD39, and displays regulatory function is the cortical positively selected DP population (27, 28, 36). The thymic cellular populations and signals mediating their positive selection and concomitant recruitment to the Treg lineage may include cortical TECs and macrophages, as well as IL-2/IL-15, shown by immunohistochemistry to be expressed in the human thymic cortex (39). The DP FOXP3^+^ thymocyte compartment directly correlates with the CD4SP FOXP3^+^ subset, denoting a precursor–product relationship (36). Upon interaction with activated pDCs, post-selection DP FOXP3^neg^ cells may also differentiate into Tregs, in a costimulation-dependent manner (38). Medullary CD4SP FOXP3^neg^ thymocytes can also acquire FOXP3 expression upon cognate interaction with activated pDCs or mDCs, in a costimulation- and IL-2-dependent fashion (37, 49). In addition, CD4SP FOXP3^neg^ thymocytes may receive appropriate TCR and costimulation signals leading to CD25 acquisition and differentiation into tTreg precursors (CD4SP CD25^+^FOXP3^neg^ cells) (39). These precursors can differentiate into tTregs upon exposure to IL-2, produced mostly by cycling mature CD4SP thymocytes, or IL-15 secreted by macrophages, B lymphocytes, or mTECs (39). Whether concomitant TCR, costimulation, and γC cytokine signaling also commits medullary CD4SP FOXP3^neg^ thymocytes into tTregs, is a possibility remaining to be addressed. Finally, CD4SP CD25^neg^FOXP3^neg^ thymocytes TCR-stimulated in the presence of costimulation, TGF-β and IL-2/IL-15 signaling can also acquire FOXP3 expression and differentiate into CD4SP tTreg (19, 39).

## The Unique Properties of Human CD8SP tTregs

In addition to the CD4SP human tTregs, a CD8SP population with phenotypic and functional Treg characteristics is present in the human thymus. CD8SP human tTregs express several Treg-associated markers, such as FOXP3, CD25, CTLA-4, and GITR, although at lower levels than CD4SP tTregs (31, 36) (Table 1). Similarly to CD4SP tTregs, they express very low levels of the IL-7 Rα/CD127 molecule (36), and are able to suppress CD4SP CD25^neg^ cells in a contact-dependent manner (31) (Table 1). A microRNA “signature” of CD8 Tregs has been recently defined in cord blood CD8 Treg (62).

Importantly, CD8SP human tTregs were found to express the α_E_ chain (CD103) of the α_E_β_7_ integrin (36), a marker associated with CD4 Tregs in the periphery (63). CD103 was also expressed at the DP stage but was very low within CD4SP thymocytes, supporting a precursor–product relationship between CD103^+^ DP and CD8SP human tTregs (36) (Table 1; Figure 1).

The role of CD103 expression on CD8SP FOXP3^+^ thymocytes remains to be addressed. High expression of E-cadherin, the main ligand of CD103, on mTEC (64) may promote retention of CD8 tTregs in the thymic medulla. Furthermore, in an *in vitro* cell adhesion assay, TEC-induced proliferation of CD8SP human thymocytes was inhibited by antibodies against E-cadherin or CD103, supporting a role for CD103 in CD8SP proliferation (64). Although the proliferation of CD8SP CD25^+^ thymocytes was not addressed in this setting, the higher levels of the active cell-cycle marker Ki67 found within CD8SP FOXP3^+^ as compared to CD8SP FOXP3^neg^ thymocytes (36) also support this hypothesis.

Despite the low frequency of CD8^+^ Treg in the steady state, they may be of special relevance in pathologic conditions [reviewed in Ref. (65, 66)]. CD103 has been associated with T-cell migration to mucosal sites (67, 68). In agreement, in the setting of colorectal cancer, CD8^+^FOXP3^+^CD25^+^ T-cells were significantly increased in peripheral blood and in colorectal cancer tissue, indicating that these cells may contribute to tumor immune escape and disease progression (69). In addition, studies in macaques have described a rapid expansion of CD8^+^FOXP3^+^CD25^+^ Tregs in the blood and colorectal mucosa following pathogenic SIV infection (70). An increase in the frequency of CD8^+^ Tregs has also been reported in HIV-1-infected patients (70).

In conclusion, a population of CD8^+^ Tregs is generated in the human thymus. A subset of post-selection DP FOXP3^+^ thymocytes expresses the tissue homing-associated molecule CD103, likely giving rise to the CD8SP FOXP3^+^CD103^+^ cells found in the medulla (Figure 1) (36). This finding supports the possibility that CD8^+^ Tregs egress the thymus expressing markers associated with mucosal homing, which may explain their very low frequency in the blood.

## Concluding Remarks

Cumulative evidence supports the existence of different pathways of Treg commitment in the human thymus that may occur at different stages of thymocyte differentiation. Their physiological contribution and the possible implications for the tTreg repertoire diversity remain unclear.

Thymus-derived Treg development does not seem to require a dedicated antigen-presenting cell population, as studies indicate that TECs, mDC, and pDC, as well as macrophages and B cells may be involved in tTreg selection. Finally, despite the proposed role of TCR signaling strength in human tTreg commitment, it has become increasingly clear that γC cytokines, particularly IL-2 and IL-15, are important mediators of lineage stabilization.

The thymus and specifically tTregs represent important therapeutic targets to manipulate tolerance in many clinical settings, namely autoimmune diseases, tumor immunity, and transplantation. Furthermore, their targeting is also critical to achieve full immunological reconstitution in primary and secondary immunodeficiencies and to decrease the morbidity associated with hematopoietic stem-cell transplantation. It is therefore of utmost importance to further investigate human tTreg development, in order to take full-advantage of the current development of immune-based therapies.

## Conflict of Interest Statement

The authors declare that the research was conducted in the absence of any commercial or financial relationships that could be construed as a potential conflict of interest.
